# Use of Vegetation Health Data for Estimation of Aus Rice Yield in Bangladesh

**DOI:** 10.3390/s90402968

**Published:** 2009-04-23

**Authors:** Atiqur Rahman, Leonid Roytman, Nir Y. Krakauer, Mohammad Nizamuddin, Mitch Goldberg

**Affiliations:** 1 NOAA-CREST, CCNY,138^th^ Street and Convent Ave, New York, NY 10031,USA; E-Mails: lroytman@optonline.net; nir@ce.ccny.cuny.edu; nizam89@gmail.com; 2 NESDIS/NOAA, 5200 Auth Rd. Camp Spring, MD 20746, USA; E-Mail: Mitch.Goldberg@noaa.gov

**Keywords:** Remote sensing, Vegetation health indices, Correlation, Principal Component Regression

## Abstract

Rice is a vital staple crop for Bangladesh and surrounding countries, with interannual variation in yields depending on climatic conditions. We compared Bangladesh yield of aus rice, one of the main varieties grown, from official agricultural statistics with Vegetation Health (VH) Indices [Vegetation Condition Index (VCI), Temperature Condition Index (TCI) and Vegetation Health Index (VHI)] computed from Advanced Very High Resolution Radiometer (AVHRR) data covering a period of 15 years (1991–2005). A strong correlation was found between aus rice yield and VCI and VHI during the critical period of aus rice development that occurs during March–April (weeks 8–13 of the year), several months in advance of the rice harvest. Stepwise principal component regression (PCR) was used to construct a model to predict yield as a function of critical-period VHI. The model reduced the yield prediction error variance by 62% compared with a prediction of average yield for each year. Remote sensing is a valuable tool for estimating rice yields well in advance of harvest and at a low cost.

## 1. Introduction

Recent floods and cyclones in South Asia have underscored the need for new sources of timely, objective and quantitative information on crop conditions. Crop growth monitoring and yield estimation can provide important information for government agencies, commodity traders and farmers in planning harvest, storage, and transportation and marketing activities [1].

Bangladesh is located between about 20° and 26° N and 88° and 92° E, in the northeast of the Indian subcontinent, and covers a total land area of 15 million hectares of which 55–65% is under cultivation. Bangladesh regularly experiences natural disasters, including floods due to heavy monsoon rainfall, droughts, and tropical cyclones. There are three seasons: a hot dry season (March to June), a warm and wet summer monsoon season (June to September) and a cool dry season (October to February) [2,11]. Annual average rainfall varies from 1,500 mm to 5,000 mm.

Rice is the staple crop and Bangladesh’s 150 million people obtain 60–70% of their calories from rice. Bangladesh, with an average crop of 40 million tons per year, is the world’s fourth largest rice producer after China, India, and Indonesia [14], and is also a rice importer. Cropping intensity is high; much productive land is double or triple cropped in rice and other crops. Three rice varieties with different seasonality and environmental sensitivity are grown: aus rice is planted before the summer monsoon and harvested in the middle of summer; aman rice is sown during the summer monsoon (July–August) and harvested November–December; and boro rice is grown over the dry season, December–January to April–May. Each of these varieties is most vulnerable to somewhat different environmental stresses. This paper will focus on interannual variability in aus rice yield.

The aus crop is either directly seeded and transplanted under rainfed and/or irrigated conditions. It is sown in March or April and harvested in late July to mid-August [14]. Aus rice phenology can be divided into three distinct phases: (1) vegetative stage (2) reproductive stage and (3) maturation stage. The vegetative phase is known as critical for aus yield; it begins at seed establishment (germination) and ends at the onset of panicle initiation.

Ground-based weather information can be employed for operational crop yield forecasts [8,9]. However, the weather station network in Bangladesh is not dense enough for efficient monitoring [2,10], prompting us to investigate the potential of using remote sensing technology. AVHRR–based vegetation health indices have been found to be very useful for early drought and flood detection and monitoring impacts on crop and pasture production around the world [6,7,9], and were shown to have predictive value for crop yield in temperate areas [12,13]. This paper investigates the application of AVHRR–based vegetation health indices for characterization of the impact of weather conditions on aus rice yield in Bangladesh.

## 2. Data and methods

**Figure 1.**
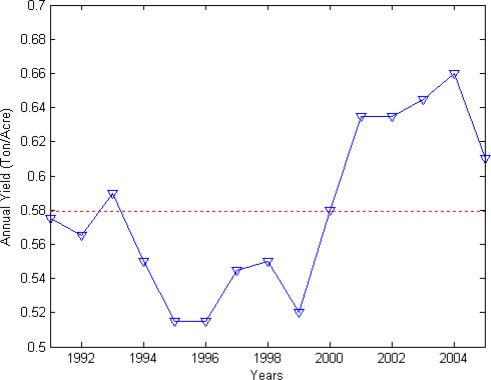
Yield of aus rice per acre in Bangladesh for 1991–2005 and its mean value (dashed line).

Aus rice statistics and satellite data for Bangladesh were used in this study. Aus rice (AR) production data were collected from the Bangladesh Bureau of Statistics [3], which estimates aus rice production and area sown from sampling surveys. Yield was calculated by dividing total AR production by the sown area. AR yield time series for 1991–2005 is shown in Figure 1.

The satellite data used were from the NOAA Global Vegetation Index (GVI) data set, which was developed by aggregating the 4 square km Global Area Coverage (GAC) daily AVHRR products to 16 square km spatial resolution and seven–day composite [6,7].

GVI is based on NDVI and BT AVHRR products, which are derived from the visible (VIS, 0.58–0.68 μm, Ch1), near infrared (NIR, 0.72–1.00 μm, Ch2) and (thermal) infrared (IR, 10.3–11.3 μm, Ch4) AVHRR channels. Post–launch–calibrated VIS and NIR intensities were converted to reflectances [6] and used to calculate the Normalized Difference Vegetation Index (NDVI = (NIR – VIS)/ (NIR + VIS)). The Ch4 counts were converted to brightness (radiative) temperature (BT) [6].

Details of the algorithm for calculating GVI from NDVI and BT are presented in Kogan [7]. Briefly, this involves: (a) elimination of high frequency noise from NDVI and BT time series, (b) estimation of the mean annual cycle, (c) calculation of multi–year climatology and (d) estimation of weekly fluctuations from the mean seasonal cycle (departure from climatology) associated with weather variations. GVI include the indices VCI characterizing plant greenness, TCI characterizing thermal conditions and VHI, a linear combination of VCI and TCI. These indices were calculated as:

(1) VCI=100(NDVI−NDVImin)/(NDVImax−NDVImin)

(2) TCI=100(BTmax−BT)/(BTmax−BTmin)

(3) VHI=a*VCI+(1−a)*TCI

where NDVI, NDVImax, NDVImin, BT, BTmax and BTmin are the smoothed weekly NDVI or BT and their 1991–2005 absolute maximum and minimum, respectively; a is the coefficient quantifying a share of VCI and TCI contribution to the VHI, which is thus a weighted average of the two. Since this share is not known for a specific location we follow the standard definition of VHI, where the shares are equal and a=0.5 (future investigation could evaluate also other combinations of VCI and TCI as possible predictors of crop yield). All three indices are scaled to range from 0 (severe vegetation stress) to 100 (exceptionally favorable conditions) [7,10].

The GVI product, at 16 km^2^ resolution, was averaged over land pixels in each of the six administrative divisions of Bangladesh. In each administrative division spatial average values of Vegetation Health Indices were calculated for each week during 1991–2005. Mean VH Indices data for the entire Bangladesh were calculated as area-weighted average vegetation health indices for the six administrative divisions.

The research strategy employed was to correlate annual yield with weekly NDVI and BT, expressed in the form of VH indices [7]. We hypothesized that there may be a strong correlation between these remotely sensed surface indicators during the early spring, i.e. around the time of the sowing and early growth of AR, and AR yields for that year. Finding and quantifying a strong correlation early in the growing season between these remotely sensed surface indicators and AR yields would allow early prediction of national AR harvest size from remote sensing, aiding farmers and consumers in decision making and providing several months’ lead time to initiate relief efforts.

## 3. Results and Discussion

**Figure 2.**
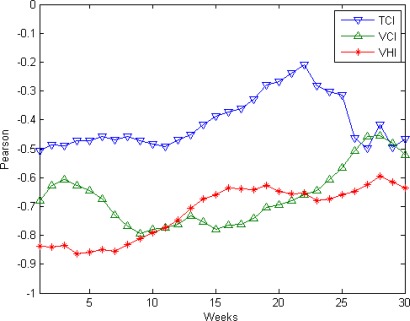
Correlation coefficient dynamics of the percent deviation of aus production from mean versus TCI, VCI and VHI.

Figure 2 shows dynamics of correlation coefficients for AR yield versus VCI, TCI and VHI for Bangladesh. Yield is highly correlated with VCI (r = −0.73 − −0.80) and VHI (r = −0.71 − −0.83) during weeks 8–13 of the year (during the period of aus rice sowing and early growth), as well as before and after. [For n=15 and assuming normally distributed data, correlation coefficients with magnitudes of 0.51 or above are significant at the 0.05 level; nonparametric (Spearman rank) regression, which is not sensitive to the distribution of the data, yields similar correlation coefficients and significance levels (not shown)]. Correlations of yield with TCI (r = −0.46 − −0.49) were also negative for weeks 8–13 but not significant at the 0.05 level.

We should note that interpretation of favorable conditions based on NDVI or VCI indices are different than the ones based on BT and TCI indices. The VCI approaches 0 (vegetation stress), when vegetation becomes less green (NDVI decreases). In opposite situation, VCI approaches 100 (favorable conditions) when vegetation becomes greener (NDVI increases). The TCI decreases, approaching 0, when weather becomes hotter (BT increases). In contrast, TCI increases, approaching 100, when weather becomes cooler (BT decreases).

**Figure 3.**
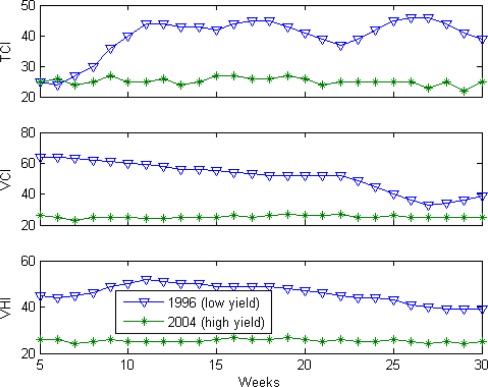
TCI, VCI and VHI for the years with the smallest and largest aus rice yield.

Differences in VCI and TCI dynamics were further investigated during the individual years with the extreme values of yield (highest and lowest). In 1996, AR yield was 0.52 ton/acre, whereas in 2004, yield was 0.66. This indicates that the 1996 (lower yield) was an unfavorable year for growing AR whereas 2004 (higher yield) was favorable. The assumption was that the environmental conditions of these years were quite different and they would be reflected in VCI, TCI and VHI values. The 1996 and 2004 VCI, TCI and VHI time series shown in Figure 3 are consistent with the observed negative correlation of yield with Vegetation Health Indices and indicate that below average AR yield is associated with higher TCI (cooler thermal condition) and VCI (high rainfall) and above average yield is associated with lower TCI (hotter) and VCI (lower rainfall) during weeks 8–13 as well as in the weeks before and after [11].

Since higher VCI and VHI imply greener vegetation and moister, cooler conditions, the negative correlation between Bangladesh aus rice yield and VCI/VHI seems counterintuitive. We hypothesize that this association is due to delayed planting, lower seed survival, and less vigorous growth of rice during relatively cool, rainy springs. In general, we expect the sign and magnitude of the correlation between vegetation indices and crop yields to vary spatially as well as across crop species and varieties, depending on what specific climatic conditions limit or favor the growth of a particular crop in a particular environmental setting.

Given these results for regression of weekly VCI, TCI and VHI individually onto yield, one possible strategy for optimally predicting AR yield would be multiple regression analysis, where the predicted yield Y_vci_ and Y_vhi_ is obtained by regression of observed yield on the linear combination of weekly VCI or VHI values. We chose VCI or VHI for weeks 8–13 as predictor variables (6 predictor variables per model) because this period corresponds to aus rice’s sowing and vegetative phase period, when it would be expected to be most sensitive to climate conditions, although correlation with crop yields of VCI and VHI in adjacent weeks is similar in magnitude (Figure 2). This would correspond to fitting the ordinary least squares (OLS) model:

(4) $$Y_{\text{vci}}=b_{0}+b_{1}*{\text{VCI}}_{8}+b_{2}*{\text{VCI}}_{9}+b_{3}*{\text{VCI}}_{10}+b_{4}*{\text{VCI}}_{11}+b_{5}*{\text{VCI}}_{12}+b_{6}*{\text{VCI}}_{13}$$

(5) $$Y_{\text{vhi}}=b_{0}+b_{1}*{\text{VHI}}_{8}+b_{2}*{\text{VHI}}_{9}+b_{3}*{\text{VHI}}_{10}+b_{4}*{\text{VHI}}_{11}+b_{5}*{\text{VHI}}_{12}+b_{6}*{\text{VHI}}_{13}$$

However, because VCI and VHI for consecutive weeks are highly correlated, we found that including VCI and VHI from multiple weeks as independent variables weakens the robustness of the correlation models. The overall models may fit the data quite well, but because the several independent variables measure similar phenomena, it is difficult to include information from each of the individual variables in a useful regression relationship. To avoid this problem, we used an alternative method of estimation, principal component regression (PCR) [12,13].

Using PCR methodology, the variables corresponding to weekly VCI and VHI for weeks 8–13 were transformed into new orthogonal or uncorrelated variables, the principal components (PCs) of the correlation matrix. In stepwise PCR, PCs were sequentially tested for their contribution to improving the regression model for AR yield, keeping only those that resulted in a significant (at the 0.05 level) reduction in residual variance. Table 1 shows summary of stepwise selection of principal components for both VCI and VHI. In each case, only the first principal component, corresponding approximately to the average value of VCI or VHI over weeks 8–13, was a significant predictor of yield.

Because the fitted model with VHI explains slightly more of the interannual variance in yield, we choose it for further evaluation. Using the final set of coefficients for variables in model equation (5) are calculated and used to develop estimation model equation (6):

(6) $$Y=119.37-0.085*{\text{VHI}}_{8}-0.079*{\text{VHI}}_{9}-0.076*{\text{VHI}}_{10}-0.072*{\text{VHI}}_{11}-0.070*{\text{VHI}}_{12}-0.068*{\text{VHI}}_{13}$$

## 4. Validation

Validation is the step in which the prediction with the chosen model is tested independently. Since the training data is short, leave-one-out cross-validation [5] (the jackknife technique) was used to verify the predictive value of vegetation indices derived from satellite imaging for rice yields later in the season. Each year of data was successively removed and a PCR model using VHI from weeks 8–13 was fit to reduced data set employing the same criteria as those used above for fitting the entire set. Finally, a prediction of AR yield for the eliminated year was made from the regression equation derived using data from the other years. As the result of this procedure, 15 independent predictions were obtained. The regression coefficients are robust to leaving out any one year of data, with the coefficients obtained staying within ∼10% or better of their value for the regression model (Equation 6) developed using the full data set.

**Figure 4.**
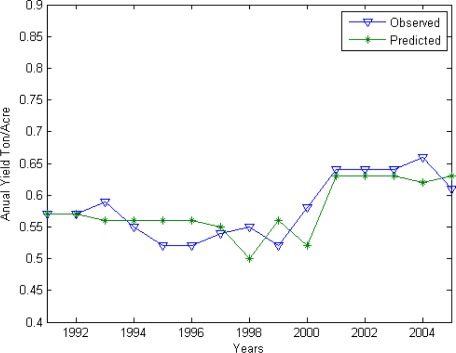
Predicted and observed aus yield for Bangladesh.

Figure 4 displays observed versus independently predicted AR yield time series, which shows that both high and low yields are generally predicted well, with R^2^ between predicted and observed yields of 0.56. The root-mean-square prediction error is 0.031 tons/acre as compared to 0.057 tons/acre for when the average yield of 0.58 tons/acre is forecast for each year, corresponding to a 62% reduction in prediction error variance.

## 5. Conclusions

In summary, three AVHRR–based VH indices characterizing surface greenness (VCI), temperature (TCI), and overall vegetation health (VHI) conditions were tested as predictors of AR yield. It was found that AR was more sensitive to vegetation health (VHI). This study shows that AR yield can be estimated from VHI index at approximately 3–4 months prior to harvest time. Weekly gridded vegetation health index data are available in real time at http://orbit.nesdis.noaa.gov/smcd/emcb/vhi.

Further investigation might include comparing the remotely sensed correlates of aus rice production in Bangladesh with those of the other two rice varieties and of other important crops; we expect that the correlation pattern between remotely sensed vegetation indices and crop yield may be quite different from crop to crop, and that the correlation pattern found will depend on crop seasonality and on growing conditions (e.g. flooded versus upland fields, and rainfed versus irrigated conditions). Similarly, this approach could be extended to predicting crop yield in other countries.

## Figures and Tables

**Table 1.** Summary of stepwise selection of VCI and VHI principal components for regression on aus rice yield.

**Indices**	**PCs**	**Model R-Square**	**Adjusted R-Square**	**F Value**	**Pr > F**
VCI	PC1	0.61	0.58	20.24	0.0006
VHI	PC1	0.62	0.59	21.49	0.0005
